# Remote Sensing and Skywave Digital Communication from Antarctica

**DOI:** 10.3390/s91210136

**Published:** 2009-12-14

**Authors:** Pau Bergadà, Marc Deumal, Carles Vilella, Joan R. Regué, David Altadill, Santi Marsal

**Affiliations:** 1 LA SALLE, Universitat Ramon Llull, Passeig Bonanova 8, 08022 Barcelona, Spain; E-Mails: mdeumal@salle.url.edu (M.D.); carlesv@salle.url.edu (C.V.); jramon@salle.url.edu (J.R.R.); 2 Grup de Geofísica, Observatorio del Ebro, Universitat Ramon Llull - CSIC, Horta Alta 38, 43529 Roquetes, Spain; E-Mails: David_Altadill@obsebre.es (D.A.); smarsal@obsebre.es (S.M.)

**Keywords:** Antarctica, remote sensing, ionosphere, geomagnetism, oblique sounding, skywave communications, DS-SS, OFDM

## Abstract

This paper presents an overview of the research activities undertaken by La Salle and the Ebro Observatory in the field of remote sensing. On 2003 we started a research project with two main objectives: implement a long-haul oblique ionospheric sounder and transmit the data from remote sensors located at the Spanish Antarctic station Juan Carlos I to Spain. The paper focuses on a study of feasibility of two possible physical layer candidates for the skywave link between both points. A DS-SS based solution and an OFDM based solution are considered to achieve a reliable low-power low-rate communication system between Antarctica and Spain.

## 1. Introduction

Antarctica is a remote and an isolated continent of large interest for the scientific community. Many research stations and remote sensors are scattered across the continent to conduct experiments related to different disciplines such as geology and physics that cannot be reproduced anywhere else on The Earth. The Research Group in Electromagnetism and Communications from LA SALLE, together with the Ebro Observatory (EO), both belonging to the Ramon Llull University, are partners of a research project for remote sensing and skywave digital communication from Antarctica. The project was born in 2003 with a double objective: transmit the data from remote sensors located at the Spanish Antarctic Station Juan Carlos I (referred to as SAS in the remainder) directly to the EO in Spain, and implement a long-haul oblique ionospheric sounder. Even though the SAS is only manned during the Austral summer, collection of scientific data is never stopped. While the station is left unmanned, the continuous set of data is stored in memory devices that will not be downloaded until the next Antarctic campaign. The information that has to be analyzed in almost real-time is transmitted to the EO in Spain through a satellite link. The skywave digital communication system is intended to transmit the information from the Antarctic sensors as a backup or even an alternative to the satellite. The oblique ionospheric sounder is a low power high frequency (HF) transceiver that monitors the ionospheric channel between the Antarctica and Spain. The sounder is fully configurable and can analyze different parameters from the ionospheric link at any frequency in the HF band. The oblique sounder is not the only sensor available at the SAS. In 1996 the EO deployed a geomagnetic observatory. The observatory is composed of two independent variometers, an Overhauser magnetometer deployed in dual axis Helmholtz coils in a *δD/δI* configuration, and a suspended tri-axial fluxgate magnetometer, along with sampling hardware and data logging software. Later, in 2004, a vertical-incidence low-power pulse-compressed ionosonde with a double delta loop antenna was also installed. The large amount of information gathered from the oblique sounding surveys is analyzed for both physics and communication purposes. One of our topics of research is to use the oblique sounding information to complement the ionospheric models obtained from the vertical sounding. From the communications point of view, the oblique sounding is used to obtain models of long-haul ionospheric channels. Parameters such as link availability, power delay profile and Doppler spread are analyzed and considered in the design of the physical layer of the skywave communication system. Currently, we have proposed two different physical layer solutions which are based on direct-sequence spread spectrum (DS-SS) signaling and orthogonal frequency division multiplexing (OFDM). Preliminary transmissions suggest that both solutions can achieve reliable low-power low-rate communications between Antarctica and Spain.

## 2. SAS Research Activity

The SAS is placed in Livingston Island (62.7 °S, 299.6 °E; geomagnetic latitude 52.6 °S) in the South Shetlands archipelago. It was inaugurated in 1988 and it is managed by the Spanish National Research Council (CSIC), which belongs to the Spanish Ministry of Science and Innovation (MICINN). Spain endorsed the Antarctic Treaty System in 1982, by which only scientific and technological issues can be developed in the Antarctica. It is MICINN who is in charge of the scientific research developed at the SAS and awards research groups grants to undertake research activities there.

The Spanish research activities in Antarctica are focused on the study of the biological and geological environment as well as the physical geography in Livingston and Deception islands and some places in the Antarctic Peninsula. Some examples of this research activities include: (i) tracking the changes in the temperature of permafrost to model the weather behaviour; (ii) study the geomorphology and movement of Johnsons-Hurd glaciers, which show a large sensitivity to temperature changes as they are very close to the melting point; (iii) study the variation of the magnetic field at Livingston Island to complete the map of the Earth's magnetic field and (iv) study the layers of the ionosphere and their relationship with the Sun activity Many of these research projects collect data of temperature, position, magnetic field, height, *etc.* that are stored in data loggers. Also some of the data are transmitted to research laboratories in Spain.

In this project, we work on the design of a system for oblique ionospheric sounding and to transmit the data from remote sensors located at the SAS to Spain. The situation faced in this research project is similar to other situations where communication is scarcely possible due to economic or coverage problems. Therefore, the solutions and conclusions presented in this work may be adopted in other situations such as communications in developing countries or communications in the middle of the ocean.

## 3. Remote Sensors at the SAS

In this section we describe the main sensors located at the SAS, including a geomagnetic sensor, a vertical incidence ionosonde and a oblique incidence ionosonde. The geomagnetic sensor and the vertical incidence ionosonde are commercial solutions from third parties independent of this project. The oblique incidence ionosonde, which is used to sound the ionosperic channel between Antarctica and Spain, was developed by LA SALLE within the framework of this project.

### 3.1. Geomagnetic sensor

Ground-based geomagnetic observatories are intended to provide a time series of accurate measurements of the natural magnetic field vector in a particular location on the Earth's surface. The data from the worldwide network of observatories are used for several scientific and practical purposes, some examples being (i) the synthesis and updates of the global magnetic field models [1]; (ii) the study of the solar-terrestrial relationships and the Earth's space environment, framed in the modern discipline of Space Weather [2]; and (iii) the support for other types of geophysical studies in a more regional or even local framework, such as mineral exploration, plate tectonics studies [3], or risk mitigation [4].

The geomagnetic observatory at the SAS in Livingston Island participates in the global network of geomagnetic observatories [5]. It was deployed in December 1996 and it was given the three letter code LIV by the International Association of Geomagnetism and Aeronomy (IAGA). Data collection and management is driven by the EO institute. Although the Spanish station is manned only during summer season, roughly from November to February, the instruments are left recording during the whole year, which implies the equipment is unattended during more than 8 months per year.

From 1996 to 2008 the SAS observatory consisted of three huts: one houses the absolute instrument, the so-called D/I fluxgate theodolite, which permits a manual measurement of the Declination and Inclination angles of the vector magnetic field in *absolute* terms (for a discussion of the uncertainties made with this instrument see [6]). This instrument consists of a fluxgate magnetometer bar mounted on the telescope of a non-magnetic theodolite. A second hut houses a variometer of the type *δD/δI* vector magnetometer [7], which automatically measures the variations of the magnetic field vector once per minute. This instrument consists of two perpendicular pairs of Helmholtz coils, the polarization of which allows measuring the Declination and Inclination variations by means of a Proton magnetometer located at their center (see [8] for an assessment of this instrument). The Proton magnetometer, in turn, measures the total field intensity, F, when the coils are not polarized. Finally, the electronic system controlling this automatic instrument is placed in a third hut. From 2008 onward, a new variometer has been added up to the old instrumentation. The new magnetometer is a three-axis fluxgate, and allows automatically measuring the magnetic field variations from an analogue output, which is currently sampled at both, 1 and 0.1 Hz by the corresponding analog to digital converter (ADC).

Once the raw observatory data are processed, the definitive data set is sent to the World Data Centres, where the worldwide scientific community can access them. This process may well take several months; however, for several scientific and practical purposes it is convenient to have real-time access to the data, especially for those unattended and/or remote observatories such as LIV. The INTErnational Real-time MAGnetic observatory NETwork (INTERMAGNET) provides means to access the data in near real-time through a satellite link. The data are thus packed and sent to the geostationary satellites, and afterwards collected by the so-called Geomagnetic Information Nodes (GINs), where the information is freely accessible. However, almost ten years of experience with satellite transmissions have shown us that the satellite link is not 100% reliable, and therefore it is suitable to have an alternative mean to retrieve the geomagnetic data. The skywave digital communication system between Antarctica and Spain designed within the research project between LA SALLE and the EO is intended to provide this backup/alternative to the satellite link. There are two major motivations to design a backup system by skywave. One one hand, visibility problems appear when trying to reach geostationary satellites from polar latitudes, which can be overcome with a skywave link due to its higher angle of departure. On the other hand, in a not 100% reliable scenario, end-to-end reliability can be significantly increased by transmitting each frame repeatedly throughout the day, that is by using time diversity. In opposite to satellite, using time diversity in skywave does not represent an extra cost as no extra time-slots must be reserved. Moreover, in skywave the time diversity can be exploited at the physical layer by employing similar techniques to those considered in space time coding (STC). This results in larger performance improvement than if diversity is employed at the information bit level.

### 3.2. Ionosonde: Vertical incidence soundings of the ionosphere

In order to have a sensor providing ionospheric monitoring in this remote region, a vertical incidence ionospheric sounder (VIS) was installed at the SAS during 2004-2005 Antarctic survey. This ionosonde is nowadays also being used to provide information for the HF radio link employed for data transmission from the SAS to Spain. A brief description of the HF radio link and of the results obtained from it will follow after the current discussion. The VIS operating at the SAS is the Advanced Ionospheric Sounder (AIS) developed by the *Istituto Nazionale di Geofisica e Vulcanologia* (INGV) of Rome, Italy. Details of the sounder are available in [9]. Data provided by the VIS serve for conducting ionospheric research at that remote region, mainly to characterize the climatology of the ionospheric characteristics and to investigate the ionospheric effects caused under geomagnetically disturbed periods (e.g., [10, 11]). The typical pattern of the summer behavior of the ionospheric F2 characteristics and of their variability was evaluated for different Spanish Antarctic surveys, and the differences of the above patterns from survey to survey was related to the different solar activity. Such a pattern at mid-high latitudes of the SAS was also compared with the summer behavior at other mid-latitude stations and it was noticed a larger post-sunset electron density enhancement at the SAS than that recorded in other regions. The significant decrease of the critical frequency of the F2 region, foF2, and their strong uplift as observed by the significant increase in the virtual height, h'F2, for the geomagnetically disturbed period of 18 February 2005 was also investigated and explained in terms of response of the ionosphere to the geomagnetic storms [12, 14]. In addition, the data recorded with the VIS, especially the maximum usable frequency for a single hop transmission at 3,000 km reflected at the F2 layer MUF(3000), has been correlated with data extracted from the HF radio link established between the SAS and Spain [11]. The above correlations were obtained for a chain of available VIS located nearby the radio path, resulting that the frequency with the largest availability of the ionospheric channel seem to be limited by the lowest of the MUF(3000) recorded along the path and that the ionospheric conditions close to the receiver have the larger influence on the daily behaviour of the channel availability for most of the time under the investigated seasonal conditions. In [11] it is also stated that frequencies of HF radio link delivered at the receiver suffer a significant drop of power for those frequencies larger than the MUF(3000) as recorded at the receiver compared to those frequencies lower than the MUF(3000).

The above results obtained with the ionospheric sensors installed at the remote region of the SAS fit with current ionospheric research carried out by other teams for regions nearby the Antarctic latitudes and other latitudes of the globe. As a brief summary and just focusing in the more recent works, we would like to highlight the following examples which are organized according to climatological studies, meteorological events and radio HF investigations. [13, 15] discussed about the spatial correlation of the ionospheric measurements and of their implications on global ionospheric models. [16] stressed the need for long-term climatological observations of solar-terrestrial phenomena to benefit the science and applications. [17] highlighted the role of the geomagnetic activity in the secular change of the ionosphere and confirms the latitudinal dependence of the trends. [18] tracked a complete night-time Weddell Sea Anomaly (WSA) - the WSA in the ionosphere is characterized by higher plasma densities at night than during the day in the region near the Weddell Sea - and strong horizontal plasma flows that registered the high-conductivity regions of the South Atlantic Magnetic Anomaly (SAMA), and revealed that the strong east-west hemispherical difference was due to the SAMA. [19] showed that the WSA occurs only in the southern summer hemisphere for low solar activity but the WSA occurs in all seasons except for winter when solar activity is high, being most prominent during the December solstice and still strong during both equinoxes, and they conclude that WSA appears to be an extreme manifestation of the longitudinal variations. [20] also reported these anomalous diurnal variations of the ionospheric peak density NmF2 related to the WSA but not found in the diurnal variations of the ionospheric peak height hmF2. [21] showed experimental evidence that the traveling atmospheric disturbances (TADs) play significant role in the ionospheric daytime effects of geomagnetic storms and demonstrate a systematic uplifting of the ionosphere during storms. [22] found changes in the critical frequency (foF2), density (NmF2), and height (hmF2) occurring after the onset of magnetospheric convection associated with high-speed solar wind streams (HSSs) arrival at the Earth's magnetosphere. [23] suggested that impulsive Joule heating of the polar thermosphere, which was accompanied by the generation of the large-scale gravity wave packet that propagated to mid latitudes, was the most probable such source for large-scale ionospheric inhomogeneities, vertical winds monotonously increasing with increasing altitude, and upward plasma transfer. [24] presented an HF selection tool (EDAM533) that has been implemented thanks to the MUF(3000) predictions generated by real-time assimilative electron density model. Also [15] have developed an HF propagation program that takes advantage of the availability of a real-time global model of the ionosphere to specify in real time the range of frequencies that would be supported on a given HF communications circuit which favorably validates against several months of oblique propagation observations on mid latitude circuits, although its day-to-day variability does not match the observations very well, and it tends to underestimate the MUFs.

The future ionospheric research at the SAS would benefit with installation of new sensors, as a high sampling rate receiver of Global Navigation Satellite System (GNSS) signals. This type of sensor would provide information about the wave front distortion of a GNSS signal caused by electron density irregularities, which giving rise to highly transient and a random amplitude and phase modulation of the GNSS signal. Such systems have been adopted by several groups for both scientific purposes and space weather applications (e.g., [17, 25]).

### 3.3. Oblique ionosonde

**Figure 1.**
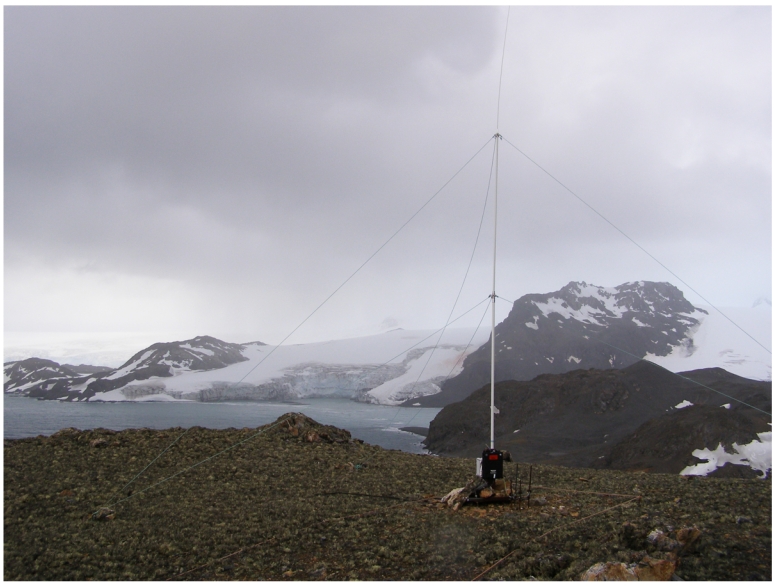
Antenna of the oblique ionosonde transmitter placed at the SAS.

**Figure 2.**
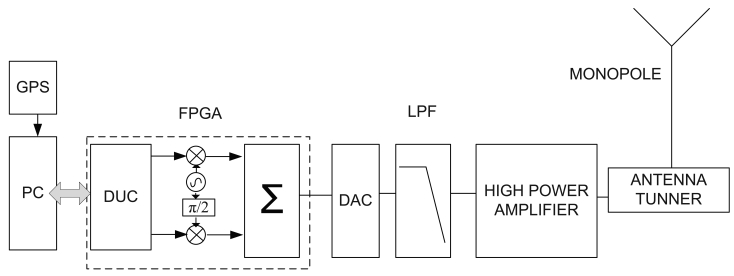

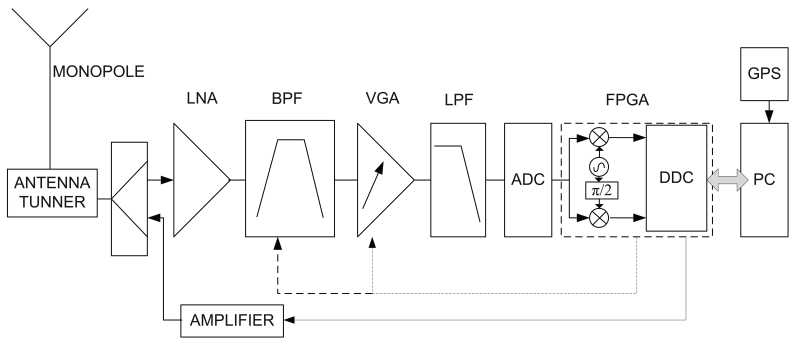
Transmitter (a) and receiver (b) system architecture based on Software Defined Radio Technology.

The aim of the oblique ionosonde [26] is to obtain the useful parameters to model the HF radiolink between the SAS and the EO. These parameters include link availability, the power delay profile of the channel and the frequency dispersion. The system performs soundings during the austral summer, coinciding with the period when the SAS is open. The sounder includes a transmitter, placed in the premises of the SAS and a receiver placed at the EO. A photography of the transmitter antenna is shown in Figure 1. Both transmitter and receiver are specifically designed to fulfill the requirements of this particular oblique sounder and the data transmitter explained in Section 4. The block diagram of the oblique sounder is shown in Figure 2.

The most relevant interest of this oblique sounder is that the link is difficult to establish. Firstly, for the distance of the link (12,700 km), which requires at least four hops to reach the receiver. And secondly, for the fact that the path crosses the equator and four different time zones.

The sounder analyzes the ionospheric radiolink each hour within a frequency band that ranges from 4.5 MHz to 16.5 MHz, with a frequency step of 500 kHz. Each sounding is divided into three intervals. The first one, with a length of 5 seconds, is used to tune the antenna. In the second one, a single tone (CW) at the frequency of interest is transmitted during 5 seconds in order to perform a narrow band sounding. And finally, during the third interval a pseudo-random noise sequence is transmitted to perform a wideband sounding. The narrowband sounding is mainly used to determine the link availability. The wideband sounding can be used to extract the parameters related with time dispersion (power delay profile) and with frequency dispersion (Doppler spread and Doppler shift). The results of this oblique soundings performed during the 2006/07 austral summer are presented in [26]. The main conclusions about link availability can be summarized as follows. (i) Between 21 UTC and 04 UTC availability is greater than 95 % at frequencies in the range 8-10 MHz,(ii) between 07 UTC and 08 UTC availability is greater than 95 % at frequencies in the range of 11-12 MHz and (iii) between 08 UTC and 09 UTC availability is greater than 95 % at frequencies in the range of 14-15 MHz. Whereas, between 10 UTC and 19 UTC availability scarcely reaches values of 20 % at any frequency. In addition, the data extracted from these oblique soundings has been correlated with the data recorded with the VIS, especially the maximum usable frequency for a single hop transmission at 3000 km reflected at the F2 layer (MUF(3000)) [11]. The above correlations were obtained from a chain of available VIS located nearby the radio path, resulting that the frequency with the best availability of the ionospheric channel seem to be limited by the lowest of the MUF(3000) recorded along the path. Moreover, the ionospheric conditions close to the receiver have a high influence on the daily behaviour of the channel availability for most of the time under the investigated seasonal conditions. In [11] it is also stated that the received signals suffer a significant drop of power for those frequencies larger than the MUF(3000) when compared with those frequencies lower than the MUF(3000).

## 4. Data Transmission

The sensors placed at the SAS premises are only manned during austral summer but they are active during summer and winter. Hence, in order to process the data in close to real time, they have to be send to the research laboratories in Spain. Since 1998, the data from the geomagnetic sensor have been transmitted by means of a geostationary satellite link from the SAS to the INTERMAGNET GIN in Edinburgh. During 2007-2008 Antarctic campaign, the new geomagnetic sensor transmitted the data to a GIN placed in Ottawa, with high reliability. The aim of this research project is to establish a low-rate low-power skywave link between Antarctica and Spain that serves as a backup or even an alternative to the satellite link.

The skywave radio link between the Antarctica and Spain has a set of characteristics and impairments that have to be taken into account to properly design the analog front-end and the digital signal processing (DSP) modules:

- The Antarctic Treaty System regulates international relations with respect to the Antarctica. Among others, it regulates the environmental laws, including aesthetic factors, and restricts the deployment of big structures because of the environmental impact. Hence, high gain antennas that would need reinforce concrete underpinning are discouraged.
- Frequency availability of this HF link is highly time dependent [26]. Therefore, the transceiver must be able to reconfigure the oscillators frequency and the antennas must cover a wide HF frequency range.
- Low transmission power is required not only to fulfill the International Telecommunication Union (ITU) recommendations but also because during austral winter, the power consumption at the SAS is restricted as it is only served by batteries charged by wind and solar power.
- The ground path between the SAS and EO is approximately 12,700 km, which corresponds to a minimum of 4 hops. A one-hop link is considered to be less than 4000 km [27].
- The attenuation of a transmitted signal at 10 MHz is of 135 dB only due to free space path loss.
- The noise and interference levels at the receiver are very high. e.g. the signal to interference level at the antenna output at frequencies between 4.5 MHz and 9 MHz can be as low as −75 dB [28].
- The ionospheric channel is a multipath channel since reflections occur at different layers of the ionosphere. Also, since the ionization level of each layer varies in time, the channel is time variant.
- A simplex ionospheric link is considered. Thus no channel state information exists at the transmitter site and no automatic repeat-request (ARQ) error control method can be implemented.

The HF band serves a wide range of applications and purposes: broadcasting, surveillance, monitoring, *etc.*, and might have a wide range of coverage. Potentially, global coverage may be reached by taking advantage of the two modes of propagation: surface wave and sky-wave propagation. So, a global organism must take care and coordinate the access to the ionospheric medium.

ITU is the leading United Nations Agency for information and communication technology issues. Since mid nineteenth century, the ITU Radiocommunication Sector (ITU-R) has coordinated the shared global use of the radio-frequency spectrum. The primary objective of the ITU-R is to ensure interference free operations of radiocommunication systems. Consequently, the world has been divided into three different regions. Region 1 comprises Europe and Africa, region 2 comprises Asia and Oceania and America forms region 3. In each region, the ITU-R suggests the attribution of a frequency band to one or several services, such as fixed, maritime mobile, aeronautic mobile, broadcasting, *etc.* When more than one service merge into the same frequency band then they are categorized into primary and secondary services. The secondary services: (i) are not allowed to interfere primary services, (ii) are not allowed to complain against primary services interferences and (iii) are not allowed to complain against previously attributed secondary services interferences. Moreover, each country has an organization to clarify ITU-R suggestions and to authorize to permanently, temporary or experimentally operate at a certain frequency.

The radio link we are dealing with, ranges through two ITU-R regions and crosses several countries. Hence a frequency spectrum request should be applied in each country, and of course it should be the same in all of them. Moreover, since the channel frequency availability is time dependent, not just one but several frequency bands along the whole HF frequency band should be requested for operation. As applying for such an international reservation of multiple frequency bands is not a feasible solution, our goal was instead to implement a communication system that should not interfere any other primary or secondary service with permanent, temporary or experimental operation authorization. To achieve it, the communication system should: (i) employ a low transmitting power, (ii) have a low power spectral density and (iii) operate with sporadic and short time transmitting periods. Also it should have a good robustness against noise, interference and time-variant multipath fading.

Several HF standards proposed by the NATO, the USA Department of Defense and the Institute for Telecomunications Science that depends on the USA Department of Commerce have been revised and we conclude that none of them satisfies the requirements of this HF link. As a result, a new physical layer solution must be proposed to match the strict requirements of the communication system considered in this project. In the following sections two preliminary physical layer proposals will be addressed.

### 4.1. System description

In this section, the transceiver used in the Antarctica to Spain HF link is described. HF receivers are typically implemented by means of several super-heterodyne conversions. Multiple intermediate frequency stages are used because of the complexity to achieve high selectivity and gain to any single frequency. However, high quality synthesizers are required and local oscillators should have to be phased locked to a reference frequency. Moreover, high quality automatic gain control systems are required in each stage to preserve the receiver performance. The aim of the transceiver architecture used in this research project is to place the ADC as close to the receiver antenna as possible, digitize the whole HF band and then, in the digital domain, select and downsample the channel of interest to baseband. This is only possible due to the development of high performance ADCs and digital signal processing (DSP) technology.

The HF transceiver developed and used in this HF link is based on a digital platform with a Field Programmable Gate Array (FPGA) from Xilinx plus a high speed digital to analog converter (DAC) and an ADC [26]. The signal is fully processed in the digital domain and therefore only amplification and some filtering are performed in the analog domain. This architecture benefits from the advantages of Software Defined Radio technology [29] and thus it allows us to implement any communication system by just introducing changes to the software. The transmitter platform, see Figure 2.a, upsamples the sampling frequency by means of a digital upconverter (DUC), filters and upconverts the baseband signal to the proper frequency that maximizes link availability. The upsampled analog signal feeds a low pass filter (LPF), then it is amplified up to 250 watts and finally delivered to a 7.5 meter monopole. In the receiver site, see Figure 2.b, the incoming signal is received with a 7.5 meter monopole, amplified with a low noise amplifier (LNA), filtered by a tunable bank of band pass filters (BPF), amplified with a variable gain amplifier (VGA) that adjusts the received power level 6 dB below the full scale range of the ADC and finally it is low pass filtered to reduce aliasing components. In the digital domain, the same FPGA digital platform used in the transmitter site mixes the received signal to baseband, filters it and decimates the sampling frequency by means of a digital down converter (DDC). Eventually, data demodulation are obtained in non real time mode after DSP.

**Figure 3.**
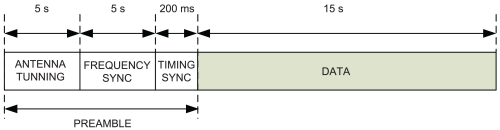
Transmitted frame.

Prior to transmit the data from the sensors, a preamble, depicted in Figure 3, must be transmitted for tuning and synchronization. Since the wide range of frequencies that maximize availability of the ionospheric link between the SAS and EO, transmit and receive antennas must have a wide frequency range of acceptable performance. Furthermore, they must be tuned to match its impedance with the transmitting and receiving elements at each different frequency. Approximately, 5 seconds are needed for antenna tuning. Clock frequency mismatch between transmitter and receiver oscillators due to time and temperature drifts must be fixed, otherwise they may have severe effects on data demodulation. During 5 seconds, a non modulated tone is transmitted and used at the receiver site to estimate the frequency deviation between transmitter and receiver clocks. This non modulated tone is also used to determine frequency availability and signal to noise ratio (SNR) statistics. The Global Positioning System (GPS) provides coarse timing synchronization between transmitter and receiver. Fine timing synchronization is acquired by means of a number of Maximal Length pseudo noise sequences of 255 chips each, transmitted at chop rate of $\frac{1}{T_{c}}=5.4\;\text{kcps}$, approximately during 200 ms. The good autocorrelation properties of these sequences assure an accurate timing synchronization.

In the following sections, two proposals to implement the physical layer of this HF radio link will be addressed. One solution is based on DS-SS signaling, the other is based on OFDM.

### 4.2. DS-SS proposal

This section is divided in three parts: (i) an introduction where DS-SS theoretic features are revised, (ii) a second one called DS-SS signaling technique where the method of modulation and demodulation used in this part of the paper is explained and compared with other techniques and finally (iii) a third one called System description and results, where the emitter and the receiver used in this experiment are described as well as the results obtained with this technique.

#### Introduction

On the basis of the sounding results described in [26], the most relevant characteristics of the long-haul ionospheric link between the SAS and Spain are: (i) negative SNR at the receiver site for a signal bandwidth of 3 kHz and the system architecture described in Section 4.1., (ii) dispersion both in time and frequency, (iii) interference. We note that spread spectrum (SS) techniques and more specifically DS-SS are a good alternative to deal with this impairments [30]. SS techniques were first developed in the military field in the middle XXth century. Nowadays it is a principal technology in wireless and mobile communications [31–33]. The application of SS techniques in ionospheric communications has also been proposed in many papers (see, for example, [35]).

DS-SS [34] is a transmission system where the signal uses a bandwidth much larger than the minimum required to send the information. Spreading is achieved by means of a code. At the receiver, a synchronized replica of that code is used to recover the data. Bit rate can be increased by using several spreading codes to transmit different baseband symbols at the same time and frequency. This technique is known as direct sequence code division multiple access (DS-CDMA). In order to maintain the SNR per symbol constant, conventional DS-CDMA systems set the transmit power to be proportional to the number of codes that are multiplexed (assuming that no multiple access interference occurs). However, as previously described the transmission power in our system is fixed. Hence, the more symbols are code multiplexed the less their SNR will be. In general, in a fix-power point-to-point DS-SS based system, the SNR per symbol after despreading can be expressed as

(1) $${\text{SNR}}_{\text{d}}={\text{SNR}}_{\text{rx}}+10\log\left(\frac{G_{p}}{N_{s}}\right)\;[\text{dB}]$$

where *G_p_* is the process gain of the spreading code, *N_s_* is the number of spreading codes that are transmitted within a symbol interval and SNR_rx_ is the SNR at the input of the receiver. Note that *N_s_* = 1 in DS-SS and *N_s_* > 1 in DS-CDMA, and that (1) is defined assuming that no multiple access interference occurs, that is, the cross-correlation between all spreading codes is zero. The spectral efficiency can be expressed as

(2) $$\eta =k\cdot \frac{N_{s}}{G_{p}}[\text{bits}/\sec/\text{Hz}]$$

where *k* is the number of bits per symbol and the transmission bandwidth is assumed to be equal to the chip rate, *i.e.*, spectral outgrowth due to upsampling is not taken into account. It is interesting to notice that when the transmitted power is fixed both DS-SS and DS-CDMA have the same performance^0^, that is, if a certain SNR_d_ is desired both systems achieve the same spectral efficiency

From Equations (1) and (2) it can be observed that an increase in the spectral efficiency can only be achieved at expenses of reducing the SNR per symbol after despreading. This is in fact a major drawback for employing DS-SS/DS-CDMA in low-power skywave communication systems. Note that in such systems the SNR at the input of the receiver is very low (typically negative), therefore to achieve low bit error rate (BER), the spectral efficiency must be low.

#### DS-SS signaling technique

In the Antarctica to Spain HF link, when a transmission bandwidth of 3 kHz is used, the typical SNR at the receiver is SNR_rx_ = −5 dB [26]. Preliminary transmissions in [36] suggested that BPSK and *G_p_* ≥ 127 should be used. Then, assuming a 3 kHz bandwidth the maximum achievable uncoded bit rate is 23.6 bits-per-second (bps). To overcome this problem we propose a physical layer based on DS-SS signaling. DS-SS signaling consists in mapping *m* data bits into one of the *M* = (2*^m^*) spreading codes. At the receiver, the transmitted bits are obtained by correlating the received time-frequency synchronized signal with the *M* spreading codes. Maximum correlation value determines the most probable transmitted spreading code, *i.e.*, the most probable transmitted group of *m* bits. This technique was proposed to reduce the multiple access interference in DS-CDMA systems, in [37] a performance evaluation of this system employing Walsh M-ary orthogonal signaling is done. The advantages of this technique are that the spectral efficiency can be increased by hardly decreasing the SNR and that accurate channel estimation is not required. Other physical layer candidates such as conventional DS-SS and DS-CDMA require accurate channel estimation in order to avoid large performance degradation, specially if a RAKE receiver is used. The downside is that the computational complexity of the receiver is high, of order *O*(2*^m^*). It is important to notice that DS-SS and DS-CDMA achieve higher spectral efficiency by scarcely increasing the complexity at the expenses of suffering from a large degradation of the BER performance. On the other hand, DS-SS signaling achieves higher spectral efficiency by scarcely increasing the BER at the expenses of a large increase of the system complexity.

To further increase the spectral efficiency of DS-SS signaling one can modulate the spreading code by using *n* more data bits. In that sense the number of bits per spreading code is increased from *m* (DS-SS signaling) to *m* + *n* (DS-SS signaling plus modulation). The *m* bits corresponding to DS-SS signaling are obtained at the receiver as described previously, the *n* bits corresponding to the modulation are obtained as in conventional DS-SS systems assuming the spreading code estimated from the DS-SS signaling detection. Note that to detect the *n* modulation bits it is required to have an estimate of the channel response.

#### System description and results

##### Emitter

**Figure 4.**
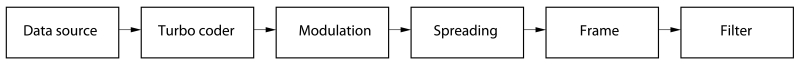

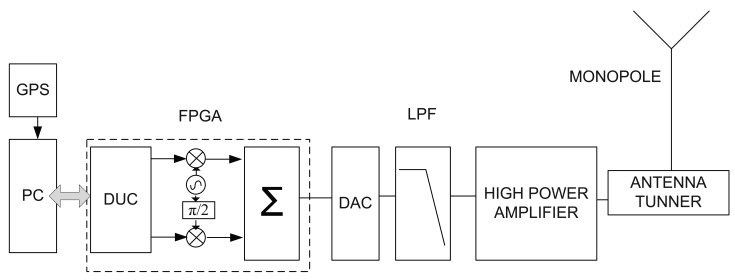
DS-SS signaling emitter (a) and receiver (b) block diagram.

The emitter block diagram is depicted in Figure 4.a. First an interleaver and a rate 1/3 turbo coder is applied to the raw data. The coded data is then divided in two blocks: one block of bits of length multiple of *n* is BPSK or QPSK modulated, the other block of bits of length multiple of *m* is used to determine the spreading code. In pure DS-SS signaling no modulation is done, *i.e., n* = 0. The transmitted frames consist of a preamble followed by the spread data multiplexed with channel estimation M-sequences. Let us recall that in pure DS-SS signaling no modulation is done, *i.e., n* = 0, therefore channel estimation is not required. Finally, the signal is filtered using a root raised cosine and delivered to the up-converters.

##### Receiver

The receiver block diagram is depicted in Figure 4.b. As previously described, fine frequency synchronization is achieved by using the preamble information. Then an impulsive noise limiter is used to improve the receiver performance. Channel estimation is obtained by means of correlation of the input signal and a replica of the M-sequences multiplexed in the frame. Then the signal is despread, demodulated and decoded to obtain the information data. The SNR is estimated from this data in order to evaluate the performance of the configuration under test.

##### Results

**Figure 5.**
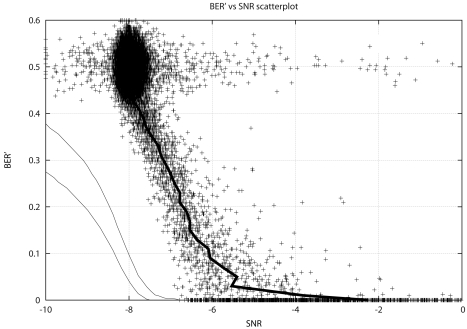
Experimental results using the following configuration: BW = 3125 Hz, 64-ary DS-SS, QPSK and rate 1/3 turbo coding. Every cross corresponds to a demodulated frame. A 3 dB loss of SNR is observed when comparing experimental and theoretical results.

An example of the experimental results is depicted in Figure 5, where the scatterplot of the BER measured in a 972 coded bits frame (BER′) versus the estimated SNR is shown. Every cross corresponds to the demodulation of a single frame. For the configuration under test, if the receiver was only limited by Gaussian noise all the crosses should lay between the two continuous lines with a 90% probability. However, most of the crosses are located outside that region, shifted to higher SNR. As expected, the implemented receiver exhibits lower performance due to mutlipath fading, nonlinear amplification, *etc.* Although the improvement of the receiver algorithms could increase the performance of the reception, a 3 dB loss of SNR is a realistic assumption when dealing with this ionospheric channel.

Many different configurations have been compared, depending on the occupied bandwidth, the sequence length (processing gain) and the modulation, and a scatterplot has been obtained for each configuration under test. On the basis of these results, we concluded that DS-SS techniques are a successful choice for data transmission in very long distance ionospheric links. We showed that a 150 bps transmission can be achieved in a 3 kHz channel bandwidth with a signal to noise ratio of −5 dB. In those conditions, more than 90 % of the packets were received without errors. This speed is sufficient for the transmission of low rate data from most of the remote sensors in the SAS.

### 4.3. OFDM proposal

The principle of OFDM is to divide a high data rate stream onto several parallel streams with lower data rate such that each sub-stream is associated with a given subcarrier. The main advantage of this concept is that each of the data streams experiences a flat fading channel, even though the overall channel is frequency selective. This means that no complex equalization at the receiver is necessary. Another advantage is that intersymbol interference (ISI) can be easily avoided by just introducing a cyclic prefix before each OFDM symbol [38]. One of the major drawbacks of OFDM is that it suffers from large peak-to-average power ratio (PAPR), which might be a major inconvenience for low power communications in long-haul ionospheric links. In the reminder of this section we present some preliminary research outcomes that try to study the feasibility of using OFDM to implement the physical layer of this long-haul HF link. Table 1 summarizes the default OFDM parameters value used throughout the experiments.

#### OFDM symbol design

One of the first aspects to clarify when designing an OFDM system is the length of the OFDM symbol and the subcarrier spacing. Since the subcarrier spacing is the inverse of the useful part of the OFDM symbol, the shorter the symbol length is the wider each subcarrier will be. Consequently the probability that each subcarrier experiences a non-flat fading increases. On the other hand, making the subcarrier spacing small to assure flat fading results in long OFDM symbols. The multipath fading channel is not constant in time, thus having very large OFDM symbols is not desirable or otherwise the channel will not be constant within an OFDM symbol.

**Figure 6.**
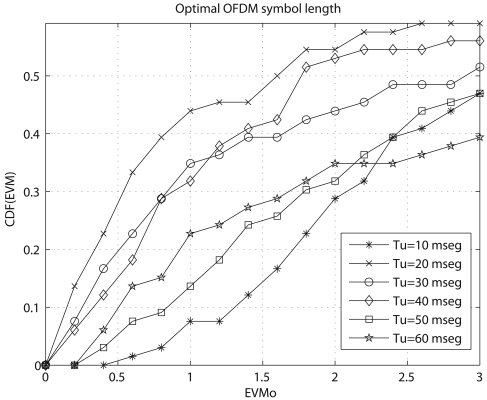
CDF of the EVM of received symbols gathered from the SAS-OE HF link. OFDM signals were transmitted at frequencies between 4.4 MHz and 17.6 MHz from 21 UTC to 09 UTC, during the 2006–2007 Antarctic survey.

To correctly design the OFDM symbol, one must take into account the coherence time of the channel, delay spread and thus coherence bandwidth of the channel. In the following, a study of the OFDM symbol design in the system performance is presented. System performance is determined by means of the error vector magnitude (EVM) of the received symbols. EVM is defined as

(3) $$\text{EV}\;M=\sqrt{\frac{P_{\text{error}}}{P_{\text{reference}}}}$$

where *P_error_* is the mean-squared error between the received constellation points and the ideally received constellation points, and *P_reference_* is the power of the outermost ideal constellation point. Figure 6 shows the cumulative density function (CDF) of EVM of real OFDM transmissions in the skywave link between the SAS and the OE. Bursts of OFDM symbols of length ranging from 10 ms to 60 ms were consecutively transmitted during several nights. As already expected, OFDM systems with too large (50, 60 ms) and too low (10 ms) symbol lengths, result in poor performance, while OFDM systems designed according to the channel characteristics (symbol lengths of 20, 30 and 40 ms) result in better performance.

#### Channel estimation

The next aspect to determine is the pilot insertion pattern for channel estimation. A correct detection of coherent OFDM systems requires that the receiver knows the amplitude and phase distortions introduced by the channel at each subcarrier. To do so, the transmitter inserts known pilot symbols in the time-frequency lattice and the receiver estimates the channel at the scattered pilot positions and then interpolates the channel on the data symbol positions. To properly estimate the channel at the data symbol positions, the pilot spacing has to fulfill the Nyquist sampling theorem. This means that the pilot separation in time, *N_T_*, and frequency, *N_F_*, must fulfill

(4) $$N_{T}\leq \left\lfloor \frac{1}{2\cdot f_{d}\cdot T_{S}}\right\rfloor \;\text{and}\;N_{F}\leq \left\lfloor \frac{1}{2\cdot \tau_{\text{max}}\cdot \Delta_{F}}\right\rfloor$$

respectively. Where *f_d_* is the Doppler frequency, *τ_max_* is the maximum delay spread of the channel and *T_S_*(*T_S_* = *T_U_* + *T_CP_*) is the OFDM symbol time. According to [26], the maximum Doppler frequency expected for this channel is always lower than 1.5 Hz and the maximum delay spread expected is always lower than 2.1 ms. Note that, *N_T_* and *N_F_* can not be arbitrarily low because both power efficiency and throughput are reduced as the pilot density increases.

During the 2005-2006 Antarctic survey, the performance of OFDM systems employing different pilot densities *(N_T_* = 5; *N_F_* = {2, 4, 8}) and pilot distributions (rectangular and hexagonal) was evaluated. Table 2 shows the performance in terms of BER for the different experiments, the throughput for each experiment is also stated. As previously discussed, one can observe that BER can be reduced by increasing the pilot density at expenses of reducing the data throughput. Also one can observe that as stated in [39], hexagonal pilot distribution always outperforms rectangular pilot distribution.

#### Envelope fluctuations and nonlinear amplification

One of the major drawbacks of OFDM is the sensitivity to nonlinear amplification. The OFDM signal is characterized by suffering from large envelope fluctuations, thus, when the time domain signal is fed through a non-linear device (such as the transmitter's power amplifier) a distortion term is introduced that results in out-of-band radiation and increased error probability. The spectral outgrowth is a limiting factor for regulated transmissions where the signal spectrum has to be located below a power spectral mask. However, our communication system is not restricted by any spectral mask and, therefore, only the BER degradation is of importance.

**Figure 7.**
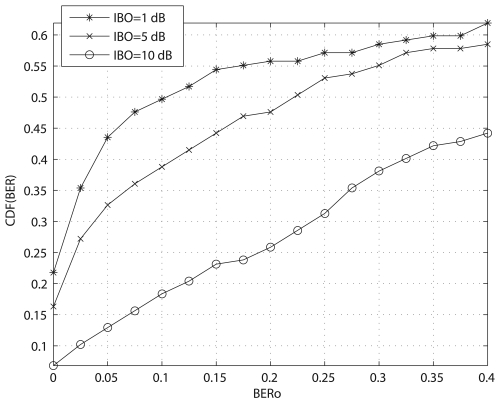
CDF of the BER of received symbols gathered from the SAS-OE HF link. OFDM signals were transmitted at frequencies between 4.4 MHz and 17.6 MHz from 21 UTC to 09 UTC, during the 2006–2007 Antarctic survey.

To reduce the nonlinear distortion introduced by the transmitter's power amplifier one can set its operating point far from saturation. In that sense the BER degradation caused by the amplifier is reduced as it is operating in a more linear region. However, one should note that, for a given amplifier, setting the operating point far from saturation means reducing the power of the transmitted signal and, as a result, reducing the SNR at the receiver. During the 2006–2007 Antarctic survey several experiments were carried out to test the effects on the distortion introduced by the transmitter's power amplifier in the overall system performance. BPSK-modulated OFDM signals with an average power 1 dB, 5 dB and 10 dB below the input saturation power of the amplifier were transmitted. As it can be observed in Figure 7, employing larger input back off's (IBO's) in the transmitter's power amplifier results in poorer BER performance, even though less nonlinear distortion is introduced. The reason is that the BER degradation caused by a nonlinearity when BPSK mapping is used is very small, even if low IBO is used. As a result, it is much convenient to place the signal close to the saturation point of the amplifier to increase the SNR at the receiver. It is worth mentioning that large modulation schemes (16-QAM or 64-QAM) are much more sensitive to the nonlinear distortion and thus one should also consider the nonlinearty effects in case of employing a 16-QAM or 64-QAM modulated OFDM system. However, this is not the case for long-haul skyvawe communications.

#### Interferences from the primary and secondary services

Interferences in the HF band are usually slow variant and have a bandwidth similar to that of our skywave communication system. As a result, if a primary or a secondary service operating at almost the same carrier frequency is present at the receiver site, the long-haul skywave communication system will be highly interfered and no communication will be possible. To avoid this one can employ a hopping pattern in the frequency domain so that the interference level is reduced.

**Figure 8.**
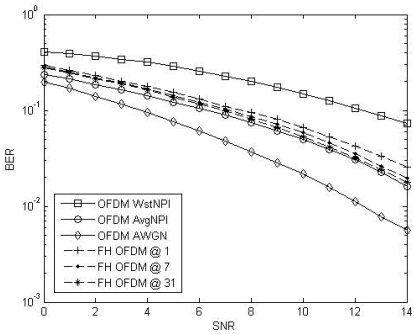
BER simulation performance of an OFDM system under the worst, the average and no interference conditions. The performance of a FH-OFDM with different hopping rates operating at the worst interference conditions is also shown.

During the 2005–2006 Antarctic campaign, noise plus interferences at the receiver site were recorded and used to test system performance under real situations. Figure 8 shows the performance of an OFDM system in a AWGN channel when such interfering signals are present. Three type of interfering conditions are evaluated: worst interference conditions (OFDM WstNPI), average interference conditions (OFDM AvgNPI) and no interference (OFDM AWGN). The performance of an OFDM system employing frequency hopes of 1 kHz every 1, 7 and 31 OFDM symbols in the worst interference conditions is also shown. As it can be observed, interferences cause a serious impact on the system performance. But it can be reduced by introducing frequency hopping. For instance, as it can be observed an OFDM system employing frequency hopes of 1 kHz every 31 symbols operating in the worst interference conditions has almost the same performance as a conventional OFDM system operating at average interference conditions. When comparing the different hopping rates, we observe that a better performance is achieved for slow frequency hopes. The reason is that when the hopping rate increases, less pilots are used to interpolate the channel in each block, yielding to a larger channel interpolation error and, thus, poorer system performance.

## 5. Conclusions

In this paper the research work on remote sensors and data transmission undertaken by LA SALLE and the Ebro Observatory in the antarctic spanish station Juan Carlos I is revised. Three different sensors, shuch as (i) a geomagnetic sensor, (ii) a vertical ionosonde and (iii) an oblique ionosonde, are described and conclusions about research outcomes are given. These three sensors have been generating data to study the earth magnetic field in this remote region as well as the behavior of the ionosphere from a physical and from a radio communications points of view.

Moreover a proposal to implement the physical layer of the skywave link between the Antarctica and Spain is studied. The main goal of this radio link is to transmit the remote data generated by the above sensors in almost real time. Research work has been focused in two possible modulation candidates:(i) OFDM and (ii) DS-SS. Both are able to cope with the channel impairments (low SNR and high level of interference at the receiver site, multipath and time variant channel,…) and link restrictions (long distance, low transmission power, non-directive antennas,…). Both modulations can achieve a data rate of 150 bits-per-second when the channel is available, which is enough for the amount of data to be transmitted from the remote sensors.

## Figures and Tables

**Table 1.** Default OFDM parameters value.

Useful symbol length	*T_U_* = 30 ms
Cyclic prefix length	*T_CP_* = 2.8 ms
Number of subcarriers	*N_C_* = 16
Frequency pilot separation	*N_F_* =8
Time pilot separation	*N_T_* =5
Channel estimation method	Least squares
Interpolation method	Cubic splines
Input back off	3 dB
Modulation	Binary Phase Shift Keying (BPSK)
Error correction code	Convolutional ( $\text{code rate}=\frac{1}{2}$, constraint length = 7)

**Table 2.** BER and data throughput with different distribution and pilot spacing in the frequency domain. In time domain, pilot spacing was constant, *N_T_* = 5. Data was gathered from the SAS-OE skywave link during the 2005-2006 Antarctic survey.

Pilot distribution	Pilot density	BER without coding	Bit rate (bps)
Rectangular	*N_F_* = 8	2.9e–2	222.6
	*N_F_* = 4	1.5e–2	193.6
	*N_F_* = 2	1.2e–2	129.3

Hexagonal	*N_F_* = 8	1.6e–2	225.6
	*N_F_* = 4	1.1e–2	194.6
	*N_F_* = 2	1.0e–2	126.3
